# XB130 deficiency enhances lipopolysaccharide-induced septic response and acute lung injury

**DOI:** 10.18632/oncotarget.8326

**Published:** 2016-03-23

**Authors:** Hiroaki Toba, Tereza Tomankova, Yingchun Wang, Xiaohui Bai, Hae-Ra Cho, Zhehong Guan, Oyedele A. Adeyi, Feng Tian, Shaf Keshavjee, Mingyao Liu

**Affiliations:** ^1^ Latner Thoracic Surgery Research Laboratories, Toronto General Hospital Research Institute, Universal Health Network, University of Toronto, Toronto, Ontario, Canada; ^2^ Department of Laboratory Medicine and Pathobiology, University of Toronto, Toronto, Ontario, Canada; ^3^ Department of Surgery, University of Toronto, Toronto, Ontario, Canada; ^4^ Institute of Medical Science, Faculty of Medicine, University of Toronto, Toronto, Ontario, Canada

**Keywords:** cell death, cell proliferation, inflammatory response, septic response, transgenic mouse

## Abstract

XB130 is a novel oncoprotein that promotes cancer cell survival, proliferation and migration. Its physiological function *in vivo* is largely unknown. The objective of this study was to determine the role of XB130 in lipopolysaccharide (LPS)-induced septic responses and acute lung injury. LPS was intraperitoneally administrated to *Xb130* knockout (KO) and wild type (WT) mice. There was a significant weight loss in KO mice at Day 2 and significantly higher disease scores during the 7 days of observation. The levels of tumor necrosis factor-alpha, monocyte chemoattractant protein-1, interleukin-6 and interleukin-10 in the serum were significantly higher in KO mice at Day 2. In KO mice there were a significantly higher lung injury score, higher wet/dry lung weight ratio, more apoptotic cells and less proliferative cells in the lung. Macrophage infiltration was significantly elevated in the lung of KO mice. There was significantly increased number of p-GSK-3β positive cells in KO mice, which were mainly neutrophils and macrophages. XB130 is expressed in alveolar type I and type II cells in the lung. The expression in these cells was significantly reduced after LPS challenge. XB130 deficiency delayed the recovery from systemic septic responses, and the presence of XB130 in the alveolar epithelial cells may provide protective mechanisms by reducing cell death and promoting cell proliferation, and reducing pulmonary permeability.

## INTRODUCTION

XB130 is a novel adaptor protein for signal transduction [1, 2]. Due to its high molecular similarity with actin filament associated protein (AFAP), XB130 is also known as actin filament associated protein 1-like 2 (AFAP1L2) [3, 4]. XB130 regulates cell proliferation and cell cycle progression, prevents cell death, and promotes cell migration, through its interaction with Src protein tyrosine kinase, and its binding with p85α, the regulatory subunit of PI3K, subsequently activating the PI3K/Akt pathway [1, 3–9].

XB130 was found in colorectal cancer cells as a Src family kinase target [10], and then found as a target of RET/PTC oncogenic kinase in thyroid cancer cells [6]. XB130 protein expression was not associated with the postoperative prognosis of patients with hepatocellular carcinoma [11]. By contrary, XB130 expression was an independent prognostic factor in human esophageal squamous cell carcinoma [12]. In gastric cancer, reduced XB130 protein expression is a prognostic biomarker for shorter survival and a higher recurrence rate [13]. On the other hand, in human ductal breast cancer, XB130 was present in the cytoplasm of malignant cells, but not in the normal breast cells, and positive XB130 expression was an independent risk factor for overall survival and recurrence free survival [14]. XB130, thus, may play different role in tumorigenesis depending upon the types of cancers.

Since most of cell biology studies on XB130 were conducted in cancer cells, the physiological function of XB130 is largely unknown. Recently, there is a report that translocation of XB130 from cytoplasm to micro-filamentous structure is involved in NNK (a cigarette smoking component) induced migration of normal human bronchial epithelial cells [8]. Moreover, the interaction between XB130 and another adaptor protein, Tks5, mediates EGF and serum-induced proliferation and survival of human bronchial epithelial cells [15]. To determine the physiological function and tumorigenesis of XB130 *in vivo*, *XB130* knockout (KO) mice have been generated. XB130 deficiency could slow down the differentiation of proliferating basal cells to ciliated epithelial cells during the repair process in trachea [16]. XB130 also promotes bronchioalveolar stem cell and Club cell proliferation in small airway repair and regeneration [17]. The objective of the present study is to determine the role of XB130 in septic response and acute lung injury.

Acute lung injury (ALI) and its severe form, acute respiratory distress syndrome (ARDS), are critical and leading cause of mortality in the Intensive Care Units. In United States, there were approximately 190,000 patients of ALI/ARDS each year with approximately 40% mortality [18]. ALI/ARDS can be caused by intrapulmonary insults (such as infection, acid aspiration, ventilator-induced lung injury, ischemia-reperfusion, and etc.), as well as by extrapulmonary insults (such as sepsis, hemorrhagic shock, trauma, burn, and etc.) [19]. Lipopolysaccharide (LPS) binds to Toll-like receptor 4, and induces the production of various pro-inflammatory cytokines and development of ALI [20].

Multiple intracellular signal transduction pathways mediates ALI/ARDS [21]. For example, PI3K/Akt, mitogen-activated protein kinase, c-Jun N-terminal protein kinase and p38 activation are involved in the overproduction of reactive oxygen species, inflammatory response, cell death and alveolar epithelial cell injury [22]. Since XB130 is an upstream signal of Src, and PI3K/Akt, we used *Xb130* KO and wild type (WT) mice to determine the role of XB130 in LPS-induced septic response and ALI.

## RESULTS

### Xb130 KO mice were more susceptible to LPS-induced septic responses

**Figure 1:**
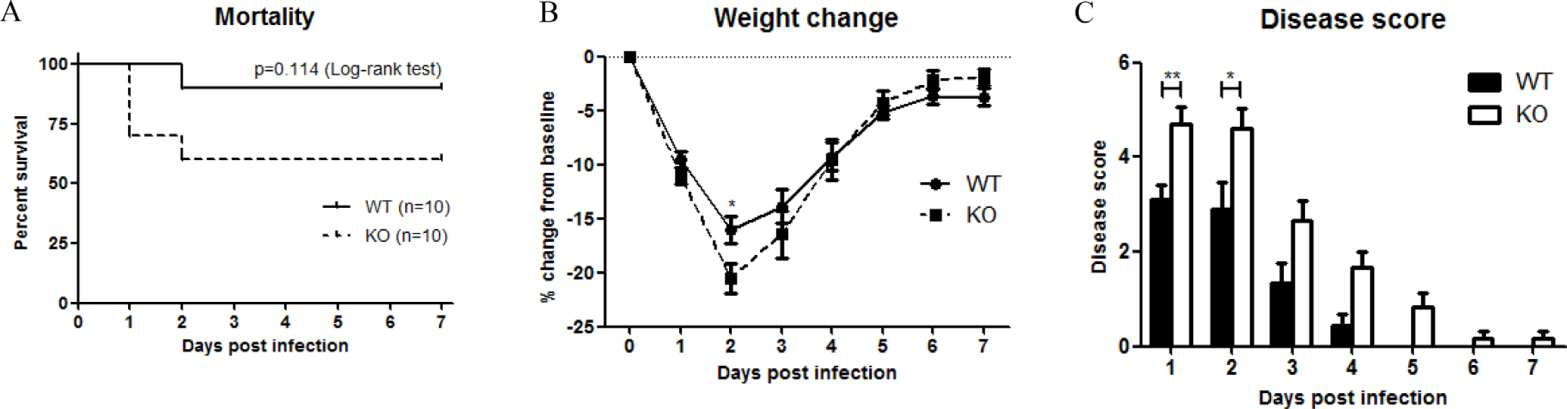
XB130 deficiency increased LPS-induced septic response in mice (**A**) Kaplan-Meier survival curves of WT mice and *Xb130* KO mice after LPS treatment (25 mg/kg). Difference in mortality was evaluated according to log-rank test (*p* = 0.114). Weight loss (**B**) and composite disease score (**C**) were recorded on different days. *p < 0.05, **p < 0.01, WT = Wild type, KO = *Xb130* knockout.

To examine the role of XB130 in LPS-induced systemic septic response, WT and *Xb*130 KO mice were challenged with 25 mg/kg LPS. Seven days' survival of *Xb130* KO mice (60%) was lower than WT mice (90%), even though it did not reach statistical significance (*p* = 0.11) (Figure 1A). *Xb130* KO mice revealed a significant weight loss at Day 2 (Figure 1B). Composite disease scores, a marker of the severity of systemic sepsis, were significantly higher in *Xb130* KO mice during the 7 days of observation period (Figure 1C).

**Figure 2:**
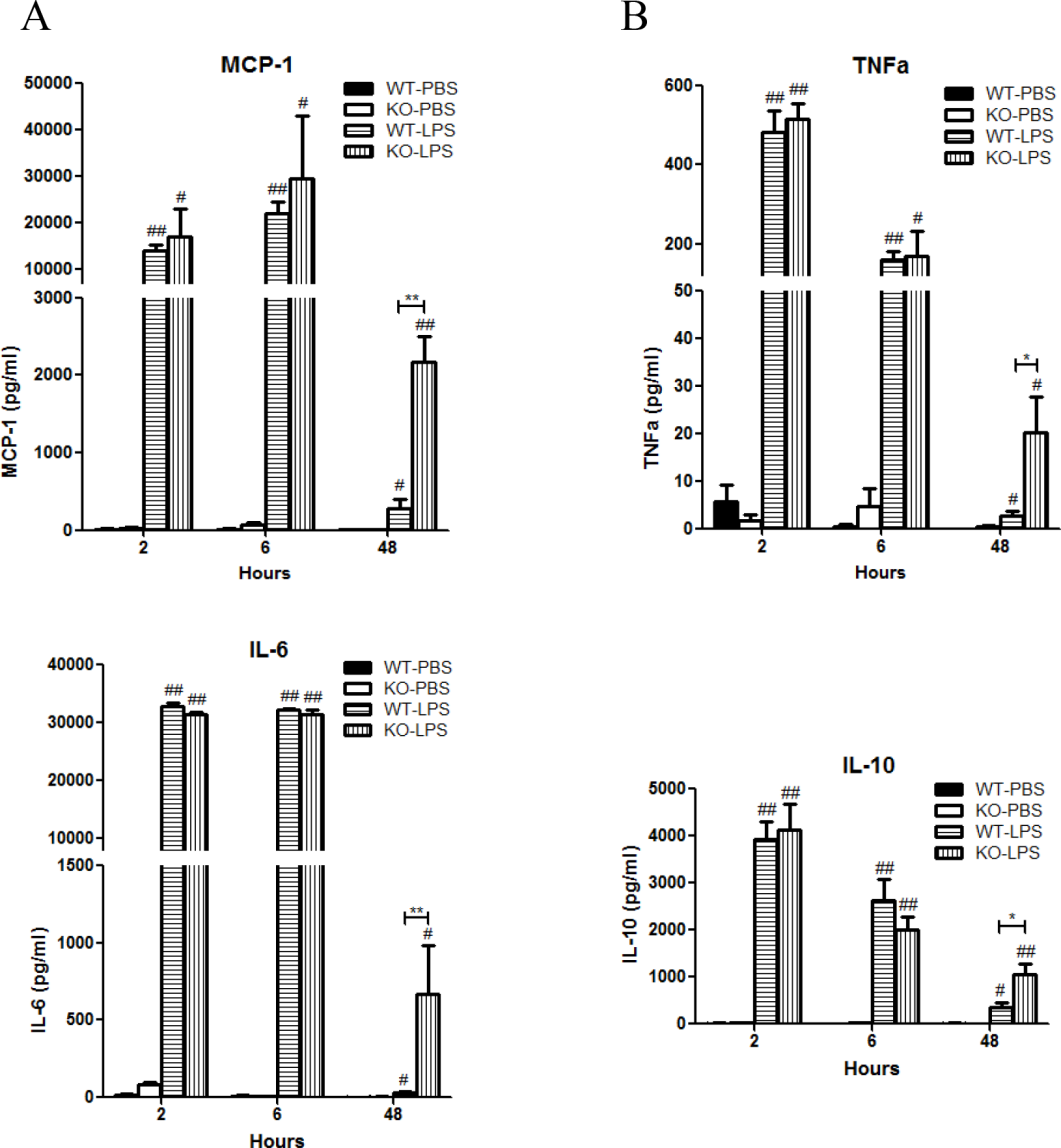
XB130 deficiency enhanced LPS-induced cytokine production (**A** and **B**) Cytokine/chemokine levels in serum were measured at 2, 6 and 48 hours after LPS treatment. *p < 0.05 and **p < 0.01 (WT vs. KO), ^#^*p* < 0.05 and ^##^*p* < 0.01 (PBS groups vs. LPS groups), WT = Wild type, KO = *Xb130* knockout.

LPS stimulation induced rapid significant increase in serum levels of monocyte chemoattractant protein-1 (MCP-1), interleukin-6 (IL-6), tumor necrosis factor-alpha (TNF-α), and IL-10 in both *Xb130* KO and WT mice (Figure 2A and 2B). It also rapidly and significantly increased serum levels of IL-1ß, IL-12p70, IL-13, IL-17 and IFNγ (Table 1). At Day 2 after LPS treatment, most of these cytokines returned to basal levels in WT mice, but the levels of MCP-1, TNF-α, IL-6 and IL-10 in *Xb130* KO mice were significantly higher compared to those in WT mice (Figure 2A and 2B).

**Table 1:** Cytokine/chemokine levels in serum

2hr (Serum)					6hr (Serum)					48hr (Serum)				
	WT-PBS	KO-PBS	WT-LPS	KO-LPS	WT-PBS	KO-PBS	WT-LPS	KO-LPS	WT-PBS	KO-PBS	WT-LPS	KO-LPS
IL-1α	243.19±85.69	170.94±46.50	224.8±29.5	154.1±18.7	324.3±135.2	184.5±52.1	200.64±39.61	206.69±32.31	147.4±46.6	275.7±119.9	67.98±24.60	103.93±22.37
IL-1β	6.55±4.55	0	122.3±15.3^#^	106.7±7.46	0	33.25±33.25	152.96±48.08	146.00±43.15	0.73±0.73	0	1.78±0.79	6.96±4.55
IL-2	5.09±4.25	0	5.36±3.59	0	0	0	0.57±0.57	0	0	0	0	0
IL-4	4.67±2.08	1.02±0.15	8.70±5.04	1.08±0.16	0.68±0.13	3.32±2.74	1.87±0.70	0.64±0.07	0.92±0.13	0.76±0.26	1.26±0.31	2.00±1.08
IL-12p70	20.23±4.64	10.11±3.17	147.7±60.6^##^	53.7±9.7^##^	5.83±5.83	37.87±36.98	69.84±18.74^#^	24.64±7.30	16.46±8.78	24.94±24.94	1.95±1.95	16.88±13.28
IL-13	30.77±14.48	0.82±0.82	96.93±76.27	22.7±9.6^#^	18.98±6.99	17.22±7.03	1.86±1.86	7.13±4.58	31.22±9.32	9.43±5.48	16.13±8.19	8.72±6.30
IL-17	10.32±4.89	4.26±0.78	32.9±14.9^**^	8.1±0.75^##^	3.22±0.82	3.77±1.21	898.16±370.78^#^	342.33±170.86^#^	2.92±0.24	2.68±1.19	3.01±0.42	5.20±0.85
IFNγ	7.77±6.03	0.44±0.44	18.19±8.54	6.9±3.01^#^	0	0	1021.71±454.44	1174.40±608.20	1.11±1.10	0	1.61±1.33	12.95±12.71

** p<0.01 (WT vs. KO)

# p<0.05

## p<0.01 (PBS vs. LPS)

### XB130 deficiency led to more severe lung injury

**Figure 3:**
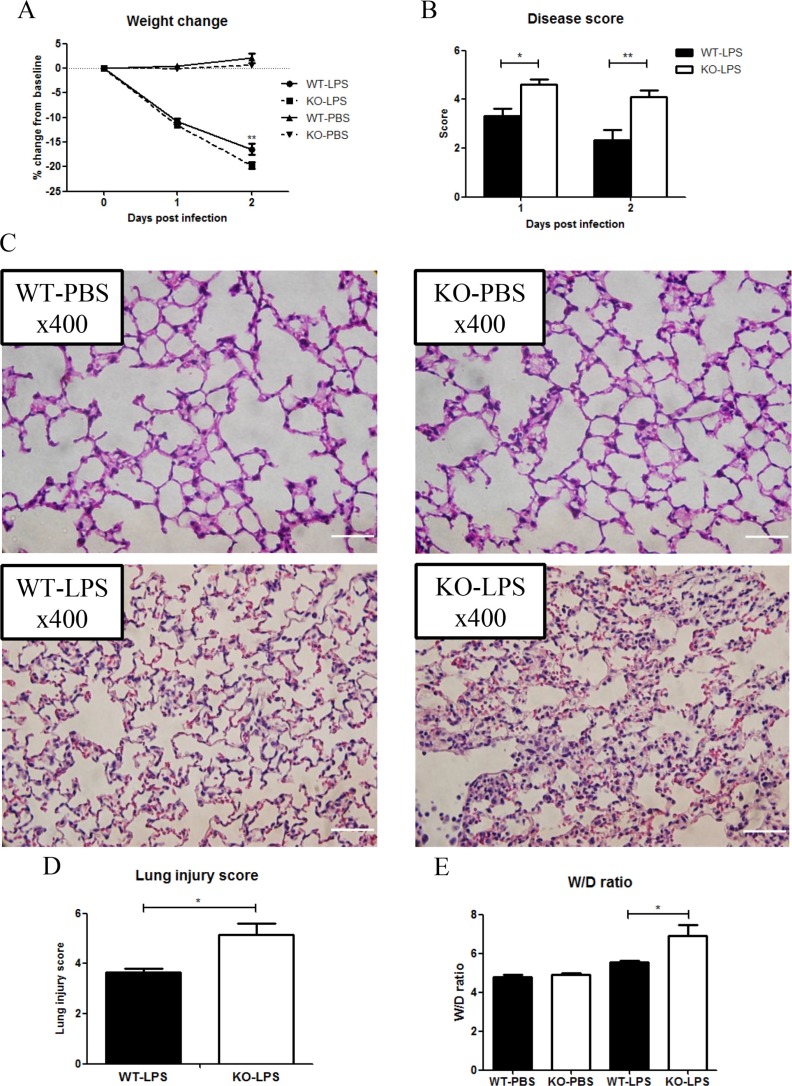
XB130 deficiency enhanced LPS-induced lung injury Weight loss (**A**) and composite disease score (**B**) were recorded for 2 days after LPS treatment. (**C**) Hematoxylin and Eosin staining showed severe lung injury in *Xb130* KO mice at Day 2 after LPS challenge. Scale Bars = 50 μm. Lung injury score (**D**) and W/D weight ratio (**E**) were significantly higher in *Xb130* KO mice. *p < 0.05, **p < 0.01, WT = Wild type, KO = *Xb130* knockout, W/D = wet/dry.

Acute lung injury is one of the severe complications of sepsis [19]. To determine the role of XB130 in LPS-induced lung injury, a separate group of animals was given LPS, or PBS as a negative control. Again, at Day 2 after LPS treatment, *Xb130* KO mice revealed a significant weight loss (Figure 3A). Significantly higher composite disease scores were found both at Day 1 and Day 2 after LPS challenge in KO mice (Figure 3B). H & E staining of lung tissue sections revealed more severe lung injury in *Xb130* KO mice at Day 2 (Figure 3C), with significantly higher lung injury score (Figure 3D) and wet/dry lung weight ratio (Figure 3E), indicating severe lung injury and pulmonary edema.

The spleens harvested after 6 hours showed normal pulp and peri-arteriolar lymphoid cells in mice that received PBS. The spleens from KO and WT mice that received LPS were congested characterized by expanded red pulp relative to the peri-arteriolar lymphoid cells. After 48 hours the spleens of LPS-treated mice in both the KO and WT group showed less congestion and appear similar to the 6 hour PBS spleens. At Day 2, the glomeruli and tubules of the renal cortices show preserved architecture. The liver parenchyma was also intact with normal portal tracts and lobular architectures. No significant differences were noted between WT and KO groups in LPS-treated animals in the spleen, kidney and liver (Supplementary Figure S1), suggesting the differences observed in the lung are organ-specific.

**Figure 4:**
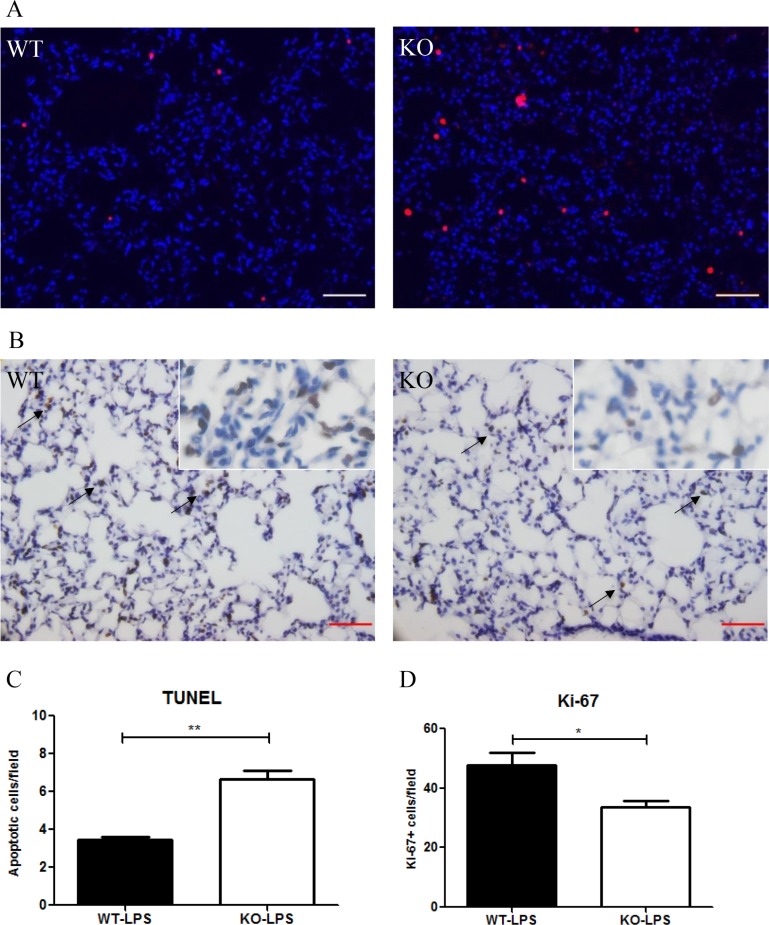
XB130 deficiency resulted in more apoptosis and less cell proliferation in LPS-induced lung injury (**A**) The number of apoptotic cells (red) was significantly higher (A–C), and the number of Ki-67+ cells (brown) was significantly lower (**B** and **D**) in *Xb130* KO mice compared to WT mice at Day 2 after LPS administration. Scale Bars = 50 μm. *p < 0.05, **p < 0.01, WT = Wild type, KO = *Xb130* knockout.

At Day 2 after LPS treatment, more numbers of apoptotic (TUNEL+) cells in the lung were observed in *Xb130* KO mice compared to WT mice (Figure 4A and 4C). By contrast, immunohistochemistry staining for Ki-67, a marker for proliferative cells, was significantly less in *Xb130* KO mice (Figure 4B and 4D).

**Figure 5:**
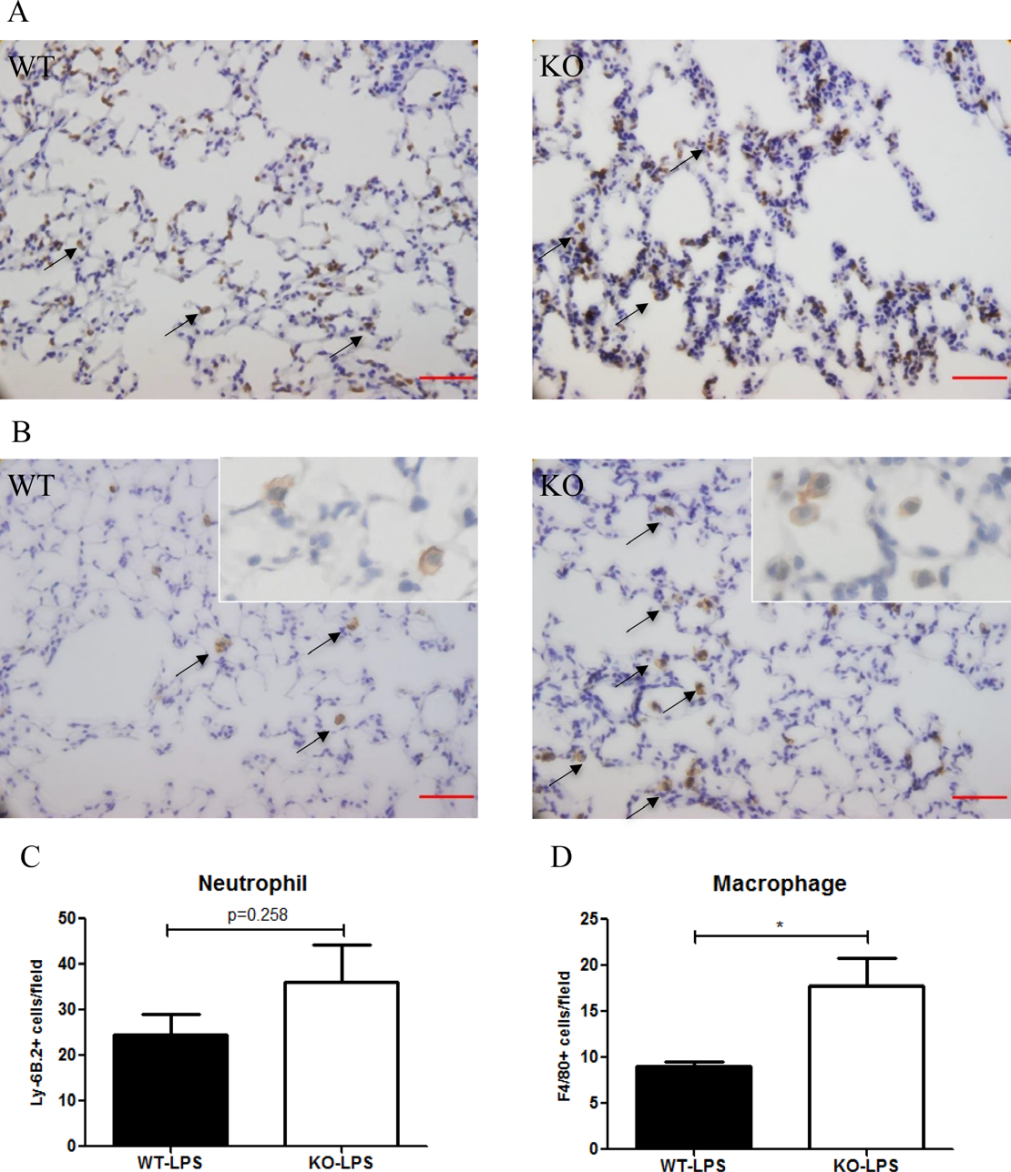
The number of neutrophils infiltrated into the alveoli at Day 2 after LPS treatment was not significantly different between WT mice and *Xb130* KO mice (A–C) On the other hand, there was significantly higher number of macrophages infiltrated into the alveoli in *Xb130* KO mice (B) (**D**). The neutrophils and macrophages were immunohistochemically stained with antibodies for Ly-6B2 and F4/80 antibodies, respectively. Scale Bars = 50 μm. *p < 0.05, WT = Wild type, KO = *Xb130* knockout.

At Day 2 after LPS treatment, the number of neutrophils (Ly-6B.2+) in the lung tissue was higher in *Xb130* KO mice (Figure 5A), but did not reach statistical significance (Figure 5C). On the other hand, the number of macrophages (F4/80+) in the lung was significantly elevated in *Xb130* KO mice compared to WT mice (Figure 5B and 5D).

**Figure 6:**
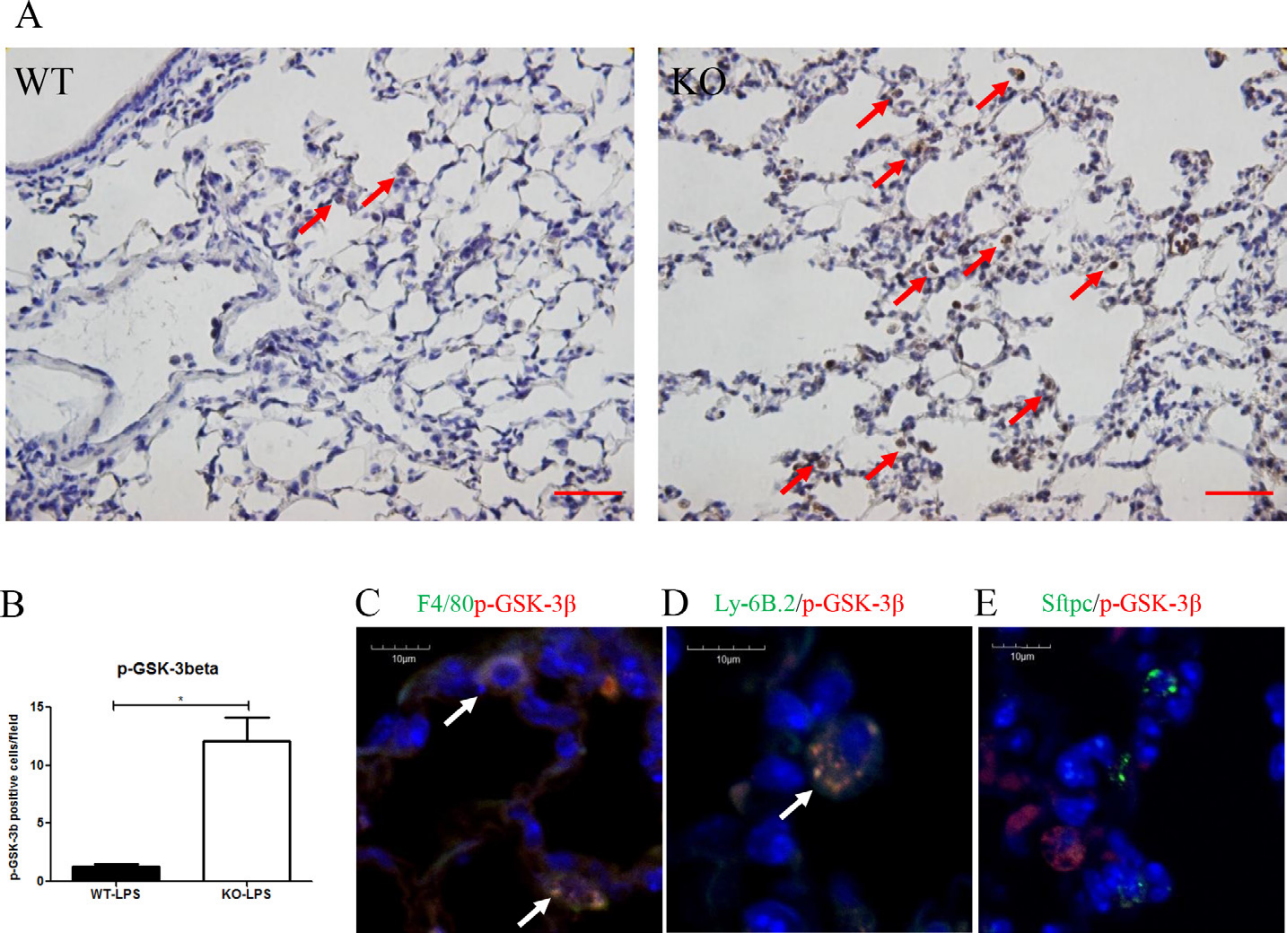
(**A**, **B**) Immunostaining for p-GSK-3β showed the number of p-GSK-3β+ cells (brown) were significantly higher at Day 2 after LPS treatment in *Xb130* KO mice compared to WT mice. Scale Bars = 50 μm. p-GSK-3β was mainly stained in neutrophils and macrophages (**C**, **D**) but not in alveolar type II cells (**E**). *p < 0.05, WT = Wild type, KO = *Xb130* knockout.

In *Xb130* KO mice, more p-GSK-3β positive cells were observed in the alveolar spaces (Figure 6A and 6B). To determine in which cell types p-GSK-3β positive staining was evident, co-IF staining for p-GSK-3β together with markers for either macrophages (F4/8), neutrophils (Ly-6B.2) or type II pneumocytes (SFTPC) was performed. Figure 6C–6E revealed that both macrophages and neutrophils, but not alveolar type II cells, were co-stained with p-GSK-3β.

### Xb130 expression was reduced in the lung after LPS administration

**Figure 7:**
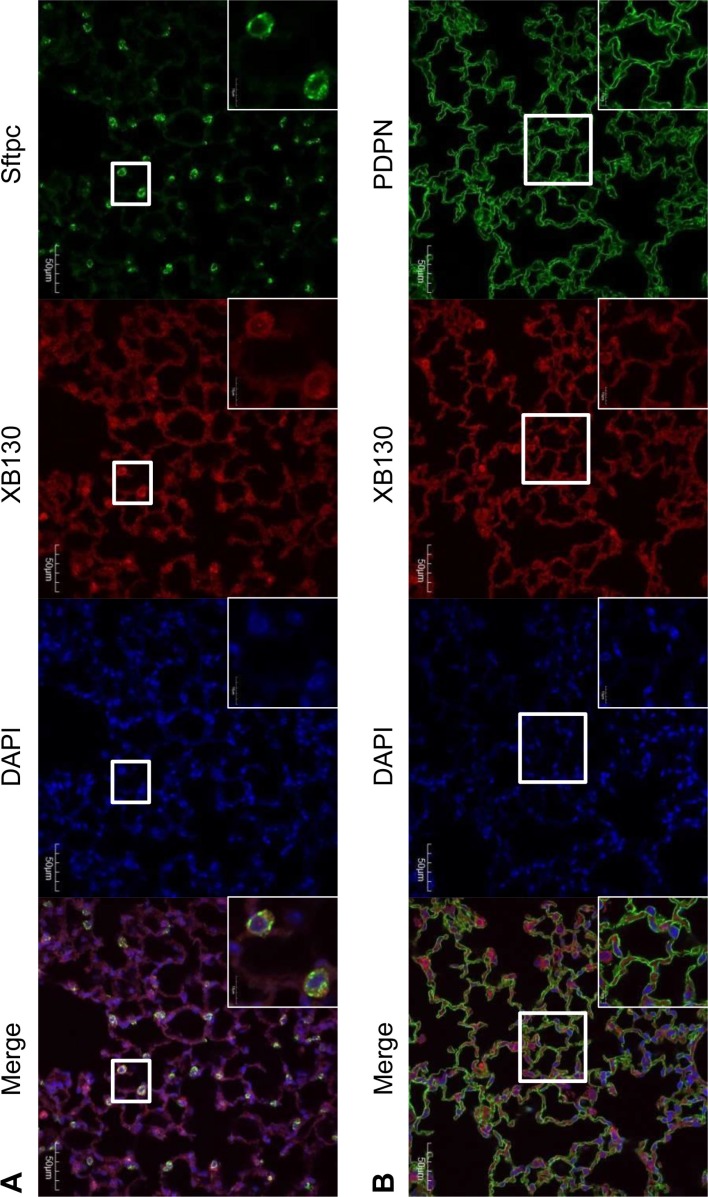
Immunofluorescence staining shows XB130 expression (XB130+: red) in alveolar type II cells (Sftpc+: green) (A) and type I cells (PDPN+: green) (B) PDPN = podoplanin, SFTPC = surfactant protein C.

XB130 is known to express in epithelial cells of various human organs [3, 9, 16]. However, the expression of XB130 has not been assessed in mouse lung yet. To detect the expression and location of XB130 in alveoli, co-IF staining for either Podoplanin (PDPN, a marker for alveolar type I epithelial cell), or Surfactant protein C (SFTPC, a marker for alveolar type II epithelial cell), together with XB130 was performed. In WT mice, XB130 was expressed in the cytoplasm of both type I and type II alveolar epithelial cells (Figure 7). As expected, no expression of XB130 was found in *Xb130* KO mice (data not shown). XB130 staining was also observed in alveolar epithelial cells in the human lung (data not shown).

**Figure 8:**
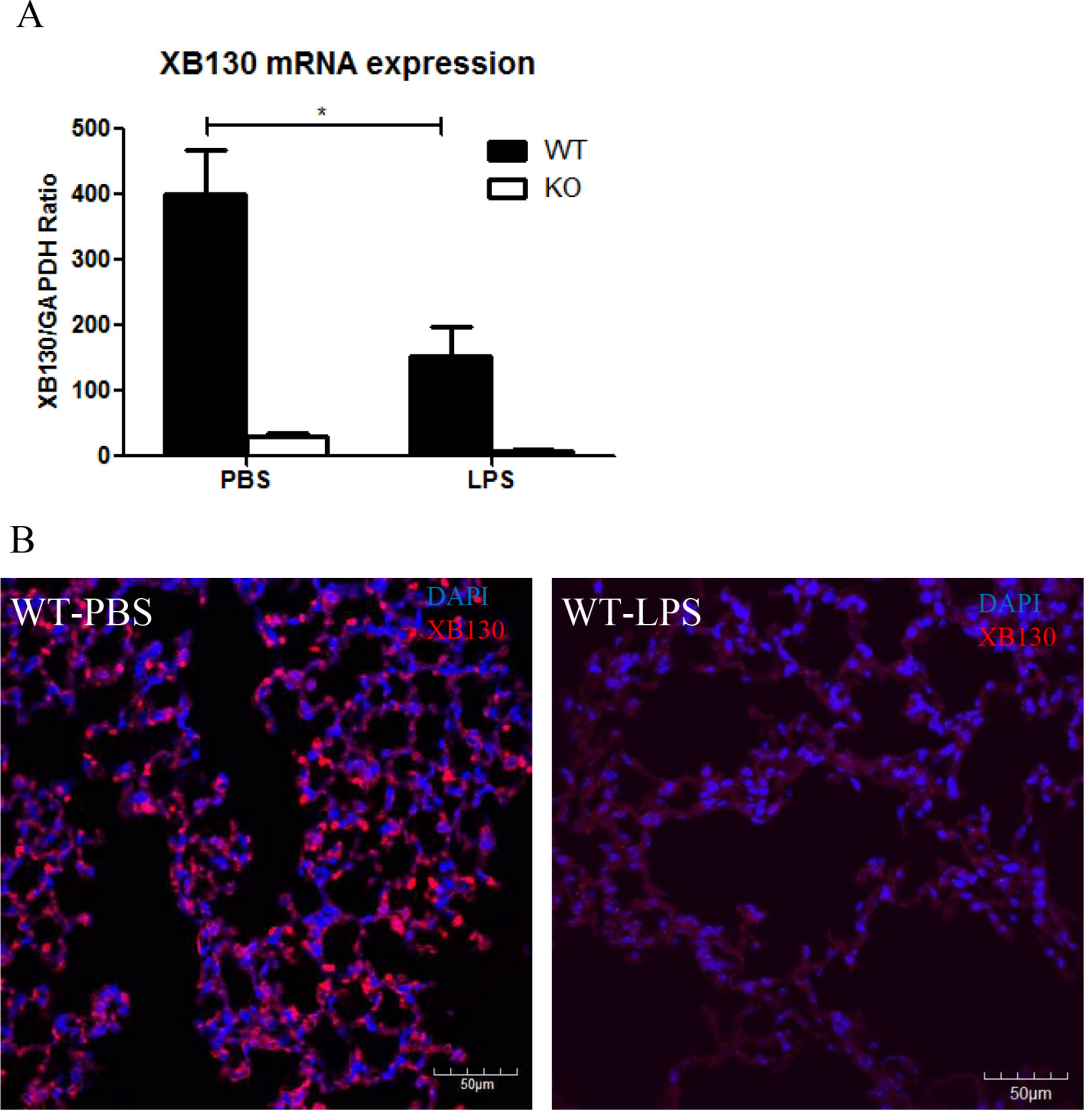
(**A**) In WT mice, *Xb130* mRNA levels were significantly reduced in the lung tissue 2 days after LPS treatment. (**B**) Immunofluorescence studies shows that the intensity of XB130 (red) expression in alveolar epithelial cells was extremely weak after LPS administration. WT = Wild type.

The *Xb130* mRNA levels were significantly lower in the lung tissue of WT mice 2 days after LPS administration than that in the PBS control group (Figure 8A). XB130 was stained in alveolar epithelial cells in the PBS control group, but very low in LPS treated group (Figure 8B).

## DISCUSSION

In this study, we showed that XB130 might play protective roles in LPS-induced systemic septic response and acute lung injury.

Endotoxin-induced septic shock is the most common cause of morbidity and mortality in intensive care units. To determine if XB130 plays a role in systemic septic response, we challenged animals with LPS intraperitoneally. We observed significant differences between *Xb130* KO and WT mice, in terms of body weight loss and composite disease score after LPS treatment. Moreover, the serum levels of several cytokines at Day 2 of LPS treatment were significantly higher in *Xb130* KO mice. MCP-1 is a CC chemokine for the recruitment of monocytes and other inflammatory cells to the sites of tissue injury and infection [23, 24]. The pro-inflammatory cytokines (TNF-α and IL-6) can activate neutrophils, which adhere to the affected capillary endothelium and migrate into air spaces, leading to severe lung injury [25, 26]. IL-10 is an anti-inflammatory cytokine and plays protective roles in controlling immunopathology during ALI [27, 28]. The expression of IL-10 can be rapidly induced by pro-inflammatory cytokines such as TNF-α [27, 28]. In this study, the levels of these cytokines in serum increased at 2 and 6 hours after LPS treatment in both groups, and then decreased in WT mice at Day 2, but remained significantly higher in the *Xb130* KO mice. The XB130 deficiency may not affect LPS challenge-induced rapid systemic inflammatory response, but may delay the recovery. At Day 2, the histology of spleen, kidney and liver appeared relatively normal, which may be due to the recovery of these critical organs from LPS challenge. The gene expression of XB130 is relatively higher in the spleen [6], which is important for immunological response. However, we did not see morphological differences between KO and WT in the spleen, kidney and liver. Therefore, why serum cytokine levels in KO mice remaining high should be further studied.

Acute lung injury is one of the most common and severe complications in septic shock [19]. In the present study, severe lung injury with more apoptotic cells, and higher W/D weight ratio and less cell proliferation were observed in *Xb130* KO mice at Day 2 after LPS treatment. Cell death and inflammation may contribute to increased vascular permeability and tissue damage [29, 30]. Xb130, as an upstream regulator of PI3K/Akt pathway, specifically binds p85α subunit of PI3K and leads to its activation [6]. Knocking down *Xb130* with siRNA in thyroid or lung cancer cells reduced cell survival, increased susceptibilities of cells to extrinsic and intrinsic signal-induced cell death, which was associated with increased cleavage of caspase-8 and caspase-9. Down-regulation of XB130 also reduced cell proliferation and cell cycle progression, together with reduced phosphorylation of p21, p27, GSK-3β and FOXO3α [3, 7, 31]. XB130 is also required for cAMP-induced amplification of IGF related mitogenicity in thyroid cells [9]. We did not specify the cell types that undergoing apoptosis or proliferation, however, we did show expression of XB130 in the alveolar epithelial cells. Thus, we would like to propose that the lack of XB130 in lung epithelial cells may contribute to apoptotic cell death in the lung after LPS stimulation, and subsequently increase the pulmonary permeability and edema. The reduced cell proliferation may explain the delayed repair of lung epithelium.

To determine whether the lack of XB130 expression affects PI3K/Akt pathway, we performed IHC staining for phosphorylated p85α, Akt and GSK-3β on lung tissues. The staining of these proteins was very weak on the alveolar wall in both WT and KO animals (data not shown). After LPS treatment, *Xb130* mRNA level was significantly reduced in the lung of WT mice, and the staining for XB130 was also extremely weak in alveolar cells. LPS and TNF-α treatment reduced XB130 protein level in cultured human bronchial epithelial cells (unpublished data); LPS may inhibit XB130 expression and reduce the protective effects of XB130 in ALI. This may also explain the weak staining of phosphorylation of p85α and Akt and GSK-3β on alveolar wall.

Interestingly, the numbers of p-GSK-3β positively stained macrophages and neutrophils were significantly higher in *Xb130* KO mice after LPS treatment. GSK-3β is a serine threonine protein kinase, and a downstream target of PI3K/Akt pathway. Human monocytes stimulated with *E. coli* LPS demonstrated increased Ser 9 phosphorylation of GSK-3β [32]. The higher numbers of p-GSK-3β positive macrophages and neutrophils may be due to prolonged injury and delayed the repair of lung epithelial cells. The accumulation of macrophages in the lung tissue in *Xb130* KO mice may contribute to the pathogenesis of ALI [33–35].

In conclusion, our results indicate that the presence of XB130 is protective to systemic septic response and related acute lung injury. Its deficiency reduces cell survival, promotes the infiltration of inflammatory cells into alveoli, increases pulmonary permeability, reduces cell proliferation, and slows down the repair process. XB130 expression can be inhibited by LPS challenge. Other ALI animal models should be used to further explore the roles of XB130. Moreover, the role of XB130 should be addressed directly in human ALI.

## MATERIALS AND METHODS

### Animal care

The study protocol was approved by the Animal Use and Care Committee of the University Health Network. All animals were housed in a pathogen-free facility, with a 12-h light-dark cycle, and they were provided with free access to water and food, and received humane care in compliance with the Guide for the Care and Use of Experimental Animals formulated by the Canadian Council on Animal Care.

*Xb130* KO mice were generated in collaboration with Dr. Tak W. Mak (University of Toronto) and maintained on a C57BL/6 genetic background. *Xb130* KO mice showed a normal life span, and did not have obvious phenotypes in a series of physiological testing compared to wild-type (WT) littermates [16].

### LPS treatment and survival analysis

Sepsis and ALI were induced in female *Xb130* KO mice and their female WT littermates (20 weeks of age) by intraperitoneal injection of LPS (25 mg/kg, *E. coli* serotype 055:B5, Sigma-Aldrich, St. Louis, MO). Two groups of animals were used with 10 mice per group: 1) WT-LPS, 2) *Xb130* KO-LPS. They were monitored for 7 days after LPS treatment for survival, body weight and composite disease score, based on the degree of ruffled fur, hunched appearance and lethargy [36]. Scores for each item were defined as 0: not present, 1: mild, 2: severe. They were added with a maximum score of 6.

### Assessment of acute lung injury and injury in other organs

Four groups of animals were used with 5 mice per group: 1) WT-PBS, 2) WT-LPS, 3) *Xb130* KO-PBS, 4) *Xb130* KO-LPS. At Day 2 after administration, mice were sacrificed, and the lung, spleen, kidney and liver were harvested. The left lung and other tissues was fixed with 4% paraformaldehyde, and the upper lobe of the right lung was used for lung wet/dry (W/D) weight study, whereas the remaining lung tissue was snap-frozen and stored at −80°C. The lung wet/dry (W/D) weight ratio was calculated by dividing the lung tissue weight before and after drying it at 80°C for 48 hours. The lung and other tissues were embedded in paraffin and cut at 5 μm thickness, and the sections were stained with hematoxylin and eosin (H & E). Images were captured using Olympus BX51-FL (Olympus Co, Ltd). We randomly chose 5 fields (×200) and assessed the lung injury with a modified score system [37]. Briefly, the alveolar edema/exudates, hemorrhage, and interstitial/alveolar cellular infiltration were scored on a scale of 1–3 (0: absent, 1: mild, 2: moderate, 3: severe) with a maximum score of 9 [38–40].

### Immunohistochemistry (IHC) and Immunofluorescence (IF) staining

The primary antibodies used were: rabbit anti-XB130 (1:1,000, Abgent, San Diego, CA), rabbit anti-Ki67 (1:100, Lab Vision, Fremont, CA), rat anti-Ly-6B.2 (1:10,000, AbD Serotec, Raleigh, GC), rat anti-F4/80 (1:500, AbD Serotec), goat anti-podoplanin (PDPN) (1:50, R & D Systems, Minneapolis, MN), rabbit anti-surfactant protein C (SFTPC, for type II cells)(1:1,000, Seven Hills Bioreagents, Cincinnati, OH) and rabbit anti-phospho-GSK-3β-Ser9 (1:500, Cell Signaling, Beverly, MA). After incubation with primary antibodies, sections were incubated with appropriate secondary antibodies. IHC was performed using a Vectastain ABC kit (Vector Laboratories, Burlington, Canada) with 3-3-diaminobenzidine as chromogen, and sections were counterstained with hematoxylin, and images were captured via Olympus BX51-FL. For IF, the secondary antibodies used were: donkey anti-goat Alexa Fluor^®^ 488, donkey anti-rabbit Alexa Fluor^®^ 555 and goat anti-mouse Alexa Fluor^®^ 555 (1:200, Invitrogen, Burlington, Canada), and sections were mounted with Prolong Gold Antifade Mountant with DAPI^®^ (Invitrogen). The slides were examined with an Olympus BX-51, and images were captured via QImaging colour camera (Olympus Co, Ltd). We randomly chose 5 fields (×200) per slide for positive cell counting.

### TUNEL assay

Cell Death was assessed by *in situ* terminal transferase dUTP nick end labelling (TUNEL) with *In Situ* Cell Death Detection Kit, TMR red (Roche, Penzberg, Germany). The slides were examined with an Axiovert 200M microscope (Zeiss, Oberkochen, Germany), and images were captured via CoolSnap HQ camera (Roper, Ottobrunn, Germany). TUNEL-positive cells were quantified from 10 randomly chosen fields per slide (× 400) using ImageJ (1.46r).

### Quantitative RT-PCR for XB130

Total RNA was extracted using RNeasy kit (Qiagen, Duesseldorf, Germany). cDNA was synthesized from total RNA using MuLV reverse Transcriptase (Invitrogen). Quantitative RT-PCR was performed using SYBR Green I master PCR kit on Light Cycler480 (Roche Diagnostics). The *Xb130* primer pairs used were 5′- TCAGCATCTCCAGAC-3′ (forward) and 5′-GGCTGTTTCCTCTCT-3′ (reverse). Each assay included a standard curve of six serial dilutions and no-template negative control. All assays were performed in triplicate. The relative expression level of *Xb130* was calculated after normalization with housekeeping gene GAPDH.

### Cytokine/chemokine measurement

For measurement of cytokine/chemokine, a separate set of animals was used with 5 mice per group. The serum was collected at 2, 6 and 48 hours after treatment, and bronchoalveolar lavage (BAL) fluid from the whole lung was collected at 48 hours after treatment as previously described [41]. Cytokines/chemokines levels in serum and cell-free BAL samples were measured using Milliplex MAP Mouse Cytokine/Chemokine Magnet Bead Panel (MCYTOMAG-70K-12, EMD Millipore, Billerica, MA).

### Statistical analysis

All values were expressed as mean ± standard error of the mean (SEM). Statistical analyses were performed by using Student's *t*-test or analysis of variance (ANOVA) with Bonferroni test as post-hoc analyses. Data were analyzed and plotted with GraphPad Prism 5.0 (GraphPad, La Jolla, CA). *P* < 0.05 was considered to be statistically significant.

## SUPPLEMENTARY MATERIALS FIGURE


