# Supplementary material for: XB130 deficiency enhances lipopolysaccharide-induced septic response and acute lung injury
Source: Oncotarget. 2016 Mar 23;7(18):25420–31. doi: 10.18632/oncotarget.8326 (PMC5041914; doi:10.18632/oncotarget.8326)
Supplement: Supplementary file 1 [file oncotarget-07-25420-s001.pdf]

XB130 deficiency enhances lipopolysaccharide-induced septic response and acute lung injury

Supplementary Materials

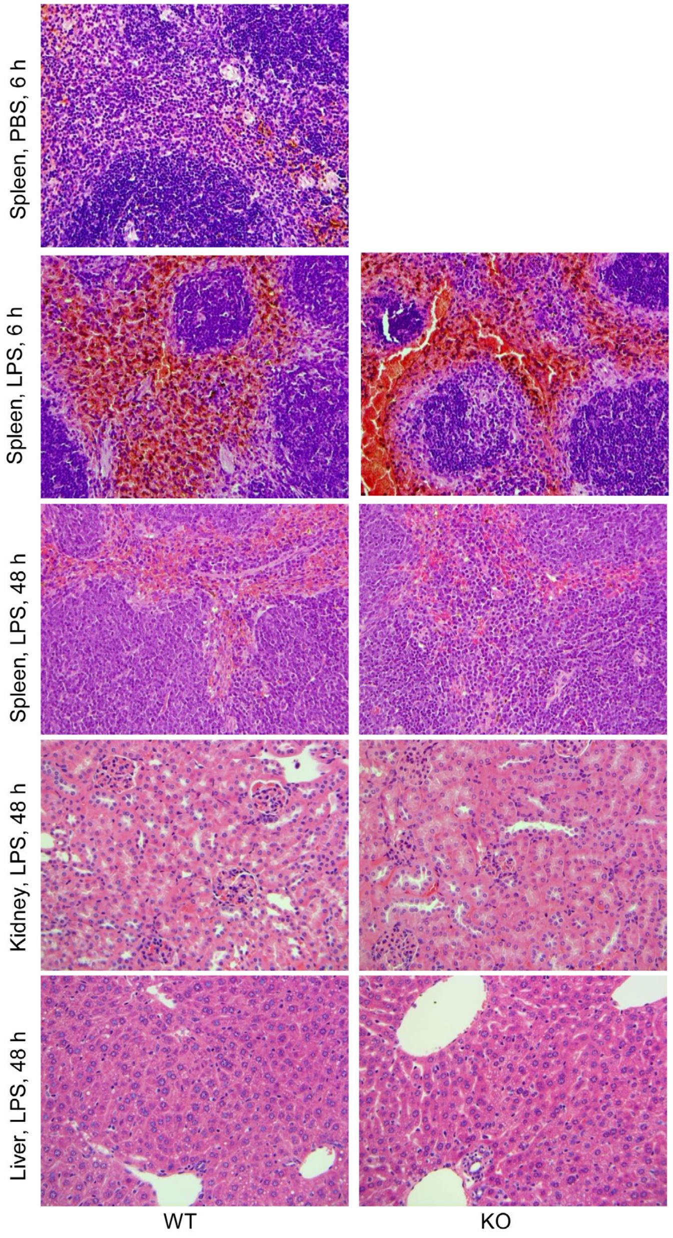

1

Supplementary Figure S1: Six hours after the challenge, spleens of PBS treated mice showed normal pulp and peri-arteriolar lymphoid cells. The spleens from KO and WT mice that received LPS were congested characterized by expanded red pulp relative to the peri-arteriolar lymphoid cells. After 48 hours of LPS treatment, spleens mice in both the KO and WT showed less congestion and appeared similar to the 6-hour PBS spleen. At 48 hours after LPS treatment, sections of the kidney (cortex), liver showed no abnormalities. No differences were observed between the WT and KO. All images were taken at magnification 200×, each represents H & E image taken from 5 mice.
